# Fascial plane blocks: from microanatomy to clinical applications

**DOI:** 10.1097/ACO.0000000000001416

**Published:** 2024-08-05

**Authors:** Carmelo Pirri, Debora Emanuela Torre, Carla Stecco

**Affiliations:** a Department of Neurosciences, Institute of Human Anatomy, University of Padova, Padua; b Department of Cardiac Anesthesia and Intensive Care Unit, Cardiac Surgery, Ospedale dell’Angelo, Venice Mestre, Italy

**Keywords:** analgesia, deep fascia, fascia, fascial blocks, injections, innervation, superficial fascia

## Abstract

**Purpose of review:**

In the last 20 years, advancements in the understanding of fasciae have significantly transformed anaesthesia and surgery. Fascial plane blocks (FPBs) have gained popularity due to their validated safety profile and relative ease. They are used in various clinical settings for surgical and nonsurgical indications. Growing evidence suggests a link between the microscopic anatomy of fasciae and their mechanism of action. As a result, knowledge of these aspects is urgently needed to better optimise pain management. The purpose of this review is to summarise the different microscopic aspects of deep/muscular fascia to expand our understanding in the performance of FPBs.

**Recent findings:**

There is ample evidence to support the role of FPBs in pain management. However, the exact mechanism of action remains unclear. Fasciae are composed of various structural elements and display complex anatomical characteristics at the microscopic level. They include various cell types embedded within an extracellular matrix abundant in collagens and hyaluronan. Increasingly, numerous studies demonstrated their innervation that contributes to their sensory functions and their role in proprioception, motor coordination and pain perception. Lastly, the diversity of the cellular and extracellular matrix, with their viscoelastic properties, is essential to understanding the FPBs’ mechanism of action.

**Summary:**

Physicians must be aware of the role of fascial microscopic anatomy and better understand their properties to perform FPBs in a conscious manner and enhance pain management.

## INTRODUCTION

In the last 20 years, advancements in the understanding of fasciae have significantly transformed anaesthesia and surgery. The development of specific fascial plane blocks (FPBs), such as interpectoral plane (IPP) block and pecto-serratus plane (PSP) block, has redefined procedures like, for example, breast and cardiac surgeries, allowing them to be performed without general anaesthesia [1,2].

FPBs, a subset of regional anaesthesia, involve injecting a local anaesthetic (LA) mixture into a plane between two fascial layers. This technique aims to spread the anaesthetic along the plane to block nerves within or crossing through it. FPBs have gained popularity due to their validated safety profile and the relative ease with which physicians can acquire the necessary skills. Modern US imaging has expanded the number of FPBs, especially for the torso. They are used in various clinical settings for surgical and nonsurgical indications [3].

Despite their effectiveness, FPBs exhibit significant variability in clinical outcomes and anatomical spread of the injectate. The sensory blockade's dermatomal distribution often does not align with the expected innervation, showing variability among patients and even within the same patient on different sides. Nevertheless, FPBs generally provide reliable motor-sparing and opioid-sparing analgesia [1,2,4^▪^]. The exact mechanism of action remains unclear and likely involves more than just the spread of LA around nerves. Indeed, fasciae, rich in nerve endings and receptors, may play a role in proprioception and pain perception. FPBs use the unique microscopic structure of fascia, which is critical for understanding their mechanism of action. Fascia is a connective tissue comprising several microscopic elements that allow it to serve as an effective medium for local anaesthetic spread during FPBS [5]. Being fascial planes composed of various cell types, all embedded within extracellular matrix (ECM) rich in collagen fibres and hydrated glycosaminoglycans (GAGs), which aids in the movement and distribution of fluids [5], the specific patterns of cells, fibres and hyaluronan can define the movement and distribution of fluids, explain the variability among patients. Besides, with its judge innervation, the fascia itself can be a target of anaesthesia, changing the expected outcomes. Nowadays, the study of fascial tissues has advanced significantly with modern imaging techniques such as ultrasound (US) and magnetic resonance imaging (MRI), which have enhanced the understanding of their dynamic structure and alterations [6,7]. While these in vivo studies are crucial for analysing properties like thickness, stiffness and gliding, a microscopic examination is essential to fully comprehend fascial anatomy and pathology [8]. Fasciae, particularly deep muscular fascia, are described in various ways in literature [9]. Fascia is a complex, vital structure that goes beyond merely connecting tissues. It supports various physiological functions and adapts to mechanical stresses, indicating its dynamic nature [10]. Fascia is classified into several categories: superficial, deep/muscular, visceral, or meningeal. These categories can be subdivided based on anatomical location, including capsules, viscera, peritoneum and ligaments. Located directly beneath the skin, in the subcutaneous tissue, superficial fascia varies in thickness [11], more substantial in the trunk and thinning towards the extremities. Moreover, it is highly vascularised [12^▪^] and contains well developed lymphatic channels [13].

Deep/muscular fascia envelops bones, muscles, nerves and blood vessels and is characterised by its fibrous consistency and high hyaluronan (HA) content compared to other fascia types [8]. Based on their orientation, structural composition and anatomical placement, deep/muscular fasciae can be classified into two primary types: aponeurotic and epimysial fasciae [6,8]. Aponeurotic fasciae encompass all the well defined fibrous sheaths that envelope and stabilise muscle groups or provide insertion points for broad muscles, such as the deep fasciae of the limbs, the thoracolumbar fascia, and the rectus sheath in the abdomen [6,8]. While epimysial fasciae pertain to the connective tissue layers that envelop skeletal muscles and, in some instances, attach directly to the bone by periosteum, as observed in the deep trunk fascia and the epimysium surrounding the limb muscles [6,8].

The deep/muscular fascia is highly vascularised [8]. It is the primary target for fascial plane blocks (FPB), creating a potential space that becomes actualised through hydro-dissection with saline or local anaesthetics. A microscopic understanding of its cellular and molecular components is essential for advancing clinical practices.

In addition, this review will discuss the different microscopic aspects of deep/muscular fascia to expand our understanding of the performance of FPB. 

**Box 1:**
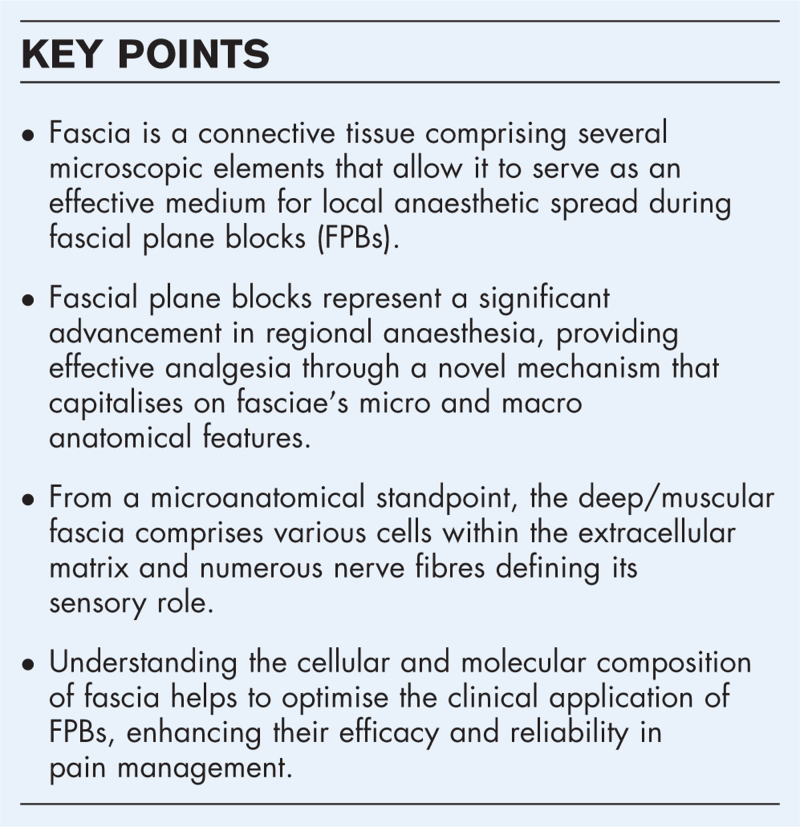
no caption available

## MICROSCOPIC STRUCTURE OF DEEP/MUSCULAR FASCIA

**FIGURE 1:**
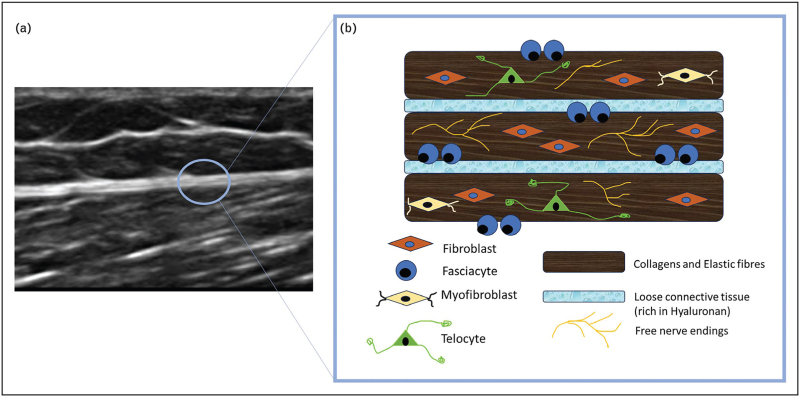
(a) Ultrasound Imaging of deep/muscular fascia, in particular fascia lata. (b) Schematic representation of microscopic anatomy of deep/muscular fascia.

From the microanatomical standpoint, the deep/muscular fascia is composed of various cells within the ECM and numerous nerve fibres that define its sensory role. The cells within the fascia adapt to different conditions, contributing to its metabolic properties and synthesising ECM. This matrix includes protein fibres (mainly collagen type I, with some type III and elastic fibres) for structural support and a water-rich ground substance rich in glycosaminoglycans, which provide flexibility, turgor, and facilitate material transport and tissue gliding (Fig. 1).

### Cellular composition

The primary cellular component of fascial tissue is fibroblasts [8]. These cells play a pivotal role in maintaining structural integrity by participating in mechanotransduction [14^▪▪^] and secreting ECM precursors [15]. Moreover, fascial fibroblasts are randomly organised and distributed in the fascial connective tissue [8]. From several studies conducted in vitro, it emerged that various stimuli could stimulate a fascial fibroblast reply. A biomechanical stimulus such as extracorporeal shockwaves (ESWS) determines the release of vesicles filled with HA and collagens [8]. Hormonal stimuli of β-oestradiol at different levels can modulate the fibroblasts’ production of certain ECM. For instance, collagen-I production decreases from 6% during the follicular phase to 1.9% during the periovulatory phase [16]. Finally, endocannabinoid stimuli can boost their production and release of HA, thereby enhancing the gliding ability of fascia [17]. The second important cellular component of fasciae is fasciacytes. They are small clusters of rounded cells located on the surface of each fascial layer, specifically designed to produce HA in ground substance [18]. These resemble fibroblasts and are positive for the fibroblast marker vimentin, but their lack of an anti-CD68 marker confirms they do not originate from monocyte/macrophage lineage. This contrasts them with other HA-producing cells, such as synoviocyte type B cells or eye hyalocytes. S100-A4 protein-positive cells serve as markers that differentiate these cells from traditional fascial fibroblasts and link them to chondrocytes, even though they do not react to the chondrocyte marker collagen II. In summary, fasciacytes represent a novel cell type specialised in HA synthesis, characterised by a rounded shape, with cytoplasm limited to the perinuclear region and possessing smaller and less elongated cellular processes [18]. About 30% of fibroblasts in healthy fascia are fasciacytes, but this can change with different stimuli. These cells are strategically located within the fascial layers and specialise in producing HA, which aids in the movement of adjacent fascial layers. Fibroblasts, on the other hand, primarily produce collagen and elastic fibres, which regulate force transmission over distances within the tissue [8].

Another important cell fasciae harbour is myofibroblasts, specialised fibroblasts with contractile abilities that modulate the tissue's basal tone [19]. Myofibroblasts density varies across different body sites, as evidenced in the human thoracolumbar fascia, which is notably higher than the plantar fascia and fascia lata (thigh) [19]. This discrepancy in density might correlate with increased instances of micro-injuries and subsequent cellular repair processes within the thoracolumbar fascia [20]. The contraction mechanism operates through adherent junctions, triggering mechanosensitive ion channels in neighbouring cells, leading to calcium influx and subsequent contraction [20]. This coordinated activity becomes particularly significant during tissue remodelling when multiple myofibroblasts contract the ECM simultaneously. This cell type is implicated in pathological fibrotic contractures affecting deep/muscular fasciae, such as Dupuytren disease [21].

Recent research has unveiled the presence of telocytes in fasciae, specialised connective tissue cells with elongated, slender extensions termed telopodes. Initially identified in the fascial lata by Dawidowics *et al.*[22], telocytes form a sophisticated network within the interstitial ECM, showcasing intricate three-dimensional communication capabilities. These cells have been detected in various fascial regions, including tensor fascia lata, crural fascia, planta fascia and thoracolumbar fascia [22–24]. While their precise functions are yet to be fully elucidated, telocytes are purported to participate in cell repair, regeneration, remodelling, immune modulation and intercellular signalling through junctions or extracellular vesicles. Consequently, their presence likely holds substantial implications for regulating myofascial pain and related disorders.

### Extracellular matrix

The fascial ECM comprises protein fibres and a water-rich gelatinous substance. The protein fibres are predominantly type I collagen, with some type III collagen and elastic fibres providing structural support and organisation [8], but there are also fibronectin, laminins and various glycoproteins. The ground substance, rich in glycosaminoglycans, gives the fascia its flexibility and enables metabolic materials transport. The diverse array of cells enveloped within this extensive fascial ECM together form a dynamic, three-dimensional network facilitating cellular residence, communication and involvement in essential functions such as growth, differentiation and migration.

Collagen fibres, notably types I and III, provide structural integrity to tissues and cells. In contrast, elastic fibres like elastin and fibrillin contribute to tissue elasticity and resilience against stretching and distension [9,10]. Variations in elastic fibre content were observed across different fascial regions, influencing tissue properties [10]. The presence and composition of collagen fibres within deep/muscular fasciae can be influenced by hormonal [16], mechanical [14^▪▪^,^15^] and chemical factors [17], impacting tissue stiffness and elasticity.

The water component of fascial ECM consists of a complex blend of GAGs, often bound to proteins to form proteoglycans and glycoproteins, with HA being a prominent GAG in fasciae [25,26]. Quantification studies by Fede *et al.*[27] revealed varying levels of HA across different deep/muscular fasciae: aponeurotic fasciae exhibited approximately 43 μg/g of HA, while epymisial fasciae showed a drastic decrease to about 6 μg/g, and the retinacula displayed an increase to 90.4 μg/g. These fluctuations correspond with the diverse gliding functions of fasciae at different anatomical sites. HA regulates fascial structure properties and influences cellular processes like proliferation, mobility, inflammation and angiogenesis, with implications for diseases such as cancer, diabetes and vascular disorders [28]. Various factors, including physical, mechanical [14^▪▪^,^15^], hormonal [16] and pharmacological influences [17], can affect the production of GAG, in HA, in the fascial ECM. Recent studies by Pirri *et al.*[14^▪▪^,^15^] demonstrated that human fascial fibroblasts can produce HA-rich vesicles in vitro following treatment with ESWS. In the same way, Fede *et al.* demonstrated [16] that these cells can produce HA-rich vesicles in vitro following treatment with CB2 receptor agonists. These vesicles enhance tissue fluidity upon release into the ECM environment, indicating a reply of these cells at different stimuli in regulating ECM formation and remodelling. HA, with its lubricating function, facilitates fascial gliding within and between densely packed collagen layers, deep fascia, muscles and muscle fibres. Its turnover rate is high due to the actions of synthetases, hyaluronidases and other degrading molecules, affecting tissue viscosity, elasticity and biological activities. The molecular size of HA influences its functions, with high molecular weight molecules exhibiting antiangiogenic and tissue repair activities, while smaller fragments display proinflammatory and proangiogenic properties [29^▪^].

Physical parameters such as temperature variations and pH levels can alter HA density, concentration, and viscosity, impacting fascial layers’ flexibility and gliding properties [28].

### Innervation of fasciae

Recent studies [30–33] revealed a significant presence of free nerve endings within the fasciae, suggesting a potential role in pain perception and regulation. Specifically, the superficial fascia in the district of the human hip exhibited the second-highest density of innervation after the skin [25].

Various sensory receptors are distributed across different types of fasciae, each with distinct functions depending on their structure and stimuli [31].

Innervation patterns within fascial tissue vary, with autonomic innervation predominant in visceral and superficial fascia; the latter share connections with skin mechanoreceptors and thermoreceptors, and deep fasciae contribute to proprioception [34]. Fascial innervation density also varies within the same fascia, with outer sublayers often exhibiting higher innervation compared to inner layers [34], but also between different topographical regions; for example, thoracolumbar fascia is more innervated than gluteal fascia [31]. Fasciae has emerged as a crucial player in pain generation. Studies have shown that hypertonic saline injections into deep/muscular fasciae result in prolonged pain duration and higher peak pain ratings than injections into subcutaneous tissue or muscle [35]. Additionally, inflammation-induced changes in the density of specific nerve fibres, such as calcitonin gene-related peptide (CGRP) and substance P (SP) – positive fibres, are observed in inflamed fascial layers [36,37]. Furthermore, endocannabinoid receptors CB1 and CB2 have been identified in various deep fasciae, indicating a potential role in pain modulation, fibrosis and inflammation [38].

## FASCIAL PLANE BLOCKS

FPBs use the unique microscopic structure of fascia, which is critical for understanding their mechanism of action. At the microscopic level, the pores between interlinked collagen fibres make fascia highly permeable to LA molecules. This permeability allows LA to traverse fascial layers without visible perforations [5]. Consequently, LA injected into a fascial plane can follow three potential pathways: firstly, they may spread and remain confined within the space of the fascial plane; secondly, they might disperse into adjacent muscle or tissue compartment through diffusion or bulk flow via larger openings; or thirdly, they could diffuse into blood vessels and be transported throughout the vascular system to distant tissue sites [5,39]. Indeed, when LAs are injected into the fascial plane, they spread through two primary processes: bulk flow and diffusion [5,39]. Bulk flow refers to the movement of the injected fluid en masse through the fascial plane, driven by the pressure of the injection. The process, known as hydro-dissection, involves the separation and expansion of the fascial layers. Factors influencing this include the injected speed, the direction of injection and the inherent elasticity of the fascia. The movement of muscles also aids in distributing the anaesthetic by continually shifting the fascial layers [5,39]. Diffusion involves the movement of anaesthetic molecules from areas of high concentration to low concentration within the fascial plane. Diffusion is facilitated by the ECM, which allows the local anaesthetic to spread and reach nociceptors and neurons within or near the fascial plane. The extent and effectiveness of diffusion are influenced by the local anaesthetic's properties and the microscopic and macroscopic features of the fascial plane that govern fluid dispersion [4^▪^,^5^,^39^]. HA's fascial content influences the fasciae's viscosity and mechanical behaviour. HA's viscosity changes with variations of temperature and pH, leading to a phenomenon termed “densification”, where the ECM becomes highly viscous, impairing the fascia's mechanical function. For instance, injecting hot saline solutions into the fascia can reduce densification, alleviating muscle stiffness and pain [40].

This variability can lead to unpredictable outcomes regarding the density and extent of sensory blockade [4^▪^,^5^,^39^]. Despite these challenges, FPBs have proven effective in relieving pain since injecting LA into the fascial plane can reduce muscle stiffness and relieve pain in myofascial syndrome patients, enhancing their range of motion [3].

Moreover, combining local and systemic analgesic effects from vascular absorption further enhances their efficacy, thanks to the fasciae's rich vascular and lymphatic network [9]. Indeed, systemic absorption of LA after FPBs may contribute to local and systemic analgesia.

Furthermore, the type and composition of fascia, whether epimysial or aponeurotic, vary according to the block type. Epimysial fasciae are characterised by thinness and fragility, whereas aponeurotic fasciae are thicker and are anchors for force transmission. Moreover, the different compositions of fascia, whether epimysial or aponeurotic, can influence LA diffusion. The capacity to confine drugs such as LA within a specific compartment is notably greater with an aponeurotic fascia compared to the epimysial fascia, which is considerably thinner and less robust. Furthermore, fascial thickness varies widely among individuals, in the same individual according to the region and level [41^▪▪^,^42^] and can present challenges for precise injections, especially considering the diameters of the needles commonly used in regional anaesthesia. Fasciae consist of multiple sublayers, and hydro-dissection during injections often reveals multiple fascial separations. HA between fascial layers facilitates dissection during injections, particularly in areas with higher HA content near joints, promoting fascial gliding and movement. Age-related changes in HA content influence fascial characteristics, with higher concentrations observed in younger individuals [43].

Additionally, anatomical variations and lines of fusion between fascial layers can complicate injections and affect the spread of LA. Scar tissue from previous surgeries or trauma may hinder LA diffusion, requiring caution during block placement. Fascial densification, which can result from various conditions, may impede LA spread but can be mitigated through interventions like massage or warm applications. Patient-related factors that change fascial ECM, such as obesity, physical status and positioning during blocks, can also influence the efficacy of FPBs [4^▪^,^5^,^39^].

These variables should be considered during block planning to optimise outcomes and minimise complications. In addition, variations in LA type, concentration and volume, and the use of adjuvants can impact block efficacy and safety [4^▪^,^5^,^39^]. To maximise the effectiveness of FPBs, several strategies can be employed:

(1) Precise injection: ensuring the LA is deposited accurately near the target area.
(2) Adequate volume: using sufficient volume to facilitate physical spread by bulk flow.
(3) Optimal concentration: adjusting the LA concentration to promote effective diffusion.

Understanding these patient-related variables and employing appropriate strategies are essential for optimal outcomes, with FPBs improving the overall analgesic effect [44].

## CONCLUSION

Fascial plane blocks represent a significant advancement in regional anaesthesia, providing effective analgesia through a novel mechanism capitalising on fasciae's micro and macro-anatomical features. While their mechanism of action continues to be studied, the combination of local and systemic effects of LA in these planes has been shown to provide substantial pain relief in various clinical scenarios. Understanding fascia's cellular and molecular composition helps to optimise the clinical application of FPBs, enhancing their efficacy and reliability in pain management.

## Acknowledgements

*None.*

### Financial support and sponsorship

*None.*

### Conflicts of interest

*There are no conflicts of interest.*

## REFERENCES AND RECOMMENDED READING

Papers of particular interest, published within the annual period of review, have been highlighted as:

▪ of special interest
▪▪ of outstanding interest
